# Impact of Monochorionicity and Twin to Twin Transfusion Syndrome on Prenatal Attachment, Post Traumatic Stress Disorder, Anxiety and Depressive Symptoms

**DOI:** 10.1371/journal.pone.0145649

**Published:** 2016-01-11

**Authors:** Berengere Beauquier-Maccotta, Gihad E. Chalouhi, Anne-Laure Picquet, Aude Carrier, Laurence Bussières, Bernard Golse, Yves Ville

**Affiliations:** 1 Department of Child and Adolescent Psychiatry, Necker-Enfants-Malades Hospital, APHP, Université Paris Descartes, Sorbonne Paris Cité, Paris, France; 2 Obstetrics and Fetal Medicine Department, Necker-Enfants-Malades Hospital, APHP, Université Paris Descartes, Sorbonne Paris Cité, Paris, France; 3 Rare Disease Center- TTTS, Necker-Enfants-Malades Hospital, APHP, Paris, France; Hospital de Especialidades del Niño y la Mujer de Queretaro, MEXICO

## Abstract

Monochronioric (MC) twin pregnancies are considered as high-risk pregnancies with potential complications requiring *in-utero* interventions. We aimed to assess prenatal attachment, anxiety, post-traumatic stress disorder (PTSD) and depressive symptoms in MC pregnancies complicated with Twin-To-Twin-transfusion syndrome (TTTS) in comparison to uncomplicated monochorionic (UMC) and dichorionic pregnancies (DC). Auto-questionnaires were filled out at diagnosis of TTTS and at successive milestones. Prenatal attachment, PTSD, anxiety and perinatal depression were evaluated respectively by the Prenatal Attachment Inventory (PAI) completed for each twin, the Post-traumatic Checklist Scale (PCLS), the State-Trait Anxiety Inventory (STAI) and the Edinburgh Perinatal Depression Scale (EPDS). There was no significant difference in the PAI scores between the two twins. In the DC and UMC groups, PAI scores increased throughout pregnancy, whilst it didn’t for TTTS group. TTTS and DC had a similar prenatal attachment while MC mothers expressed a significantly higher attachment to their fetuses and expressed it earlier. At the announcement of TTTS, 72% of the patients present a score over the threshold at the EPDS Scale, with a higher score for TTTS than for DC (p = 0.005), and UMC (p = 0.007) at the same GA. 30% of mothers in TTTS group have PTSD during pregnancy. 50% of TTTS- patients present an anxiety score over the threshold (STAI-Scale), with a score significantly higher in TTTS than in UMC (p<0.001) or DC (p<0.001). The proportion of subject with a STAI–State over the threshold is also significantly higher in TTTS than in DC at 20 GW (p = 0.01) and at 26 GW (p<0.05). The STAI-state scores in UMC and DC increase progressively during pregnancy while they decrease significantly in TTTS. TTTS announcement constitutes a traumatic event during a pregnancy with an important risk of PTSD, high level of anxiety and an alteration of the prenatal attachment. These results should guide the psychological support provided to these patients.

## Introduction

Monochorionic (MC) twin pregnancies represent 20–25% of all twin pregnancies with a continuously increasing incidence [1] [2]. Their morbidity and mortality rates are higher than in dichorionic pregnancies, because of their specific complications that include selective intra-uterine growth restriction (IUGR), intra-uterine fetal demise (IUFD) and twin-to-twin transfusion syndrome (TTTS)… The latter, also called twin oligoamnios polyhydramnios sequence, complicates around 15% of MC pregnancies. It is a hemodynamic, and hormonal, discordance secondary to imbalanced blood flow through the vascular anastomoses between the two twins and resulting in an amniotic fluid discordance. The Centre Maladies Rares-STT (Rare Disease Center- TTTS) of Necker-Enfants Malades Hospital is a national referral center for fetoscopic laser treatment of these complications. The natural history of untreated TTTS leads to intra- or perinatal death in 90% of cases [3]. The gold-standard management of TTTS consists of feto-placental surgery (fetoscopy for laser photocoagulation of placental vessels) [4]. The immediate post-operative surveillance is based on repeated ultrasound examinations at 16, 24, 36 and 48 hours post-operatively followed by weekly follow-up ultrasonographies for the rest of the pregnancy. This follow-up is pursued until 34 weeks when the patient will have a cesarean section [5] [6] [7]. This management leads to at least one twin survival rate around 88% to 92%, and a mean survival of both twins between 52% and 75% [8].

In these pregnancies, pregnant women have to cope with several announcements. In the multidisciplinary care of these patients, a psychological follow-up is offered systematically. In our clinical experience, the announcement of monochorionicity and its specific risks influences the way the woman perceives, experiences and lives her pregnancy. The pregnancy becomes a stressful event. And the women won’t be reassured until the babies are born.

As literature in this specific field is very scarce, we wanted to explore two dimensions of the psychological adjustment during these pregnancies;

- First of all we wanted to explore prenatal attachment. During a normal pregnancy, mothers deploy their representations of the unborn baby, of themselves as future mothers, of their relationship with their own parental figures, to prepare for motherhood [9] [10] [11]. In these complicated pregnancies the mother-to-be could find herself in a state of shock, unable to think or to project herself; the maternal fantasies could be blocked. During the monitoring of these pregnancies, the medical counseling becomes a consolidation of the maternal representations, giving a particular representation for each of the fetuses, the donor, the receiver, the bigger one, the smaller one, etc. [12]. Prenatal attachment is an important dimension of the parent-child relationship. It is the investment of the pregnant woman towards her unborn child. It assesses how well the mother adopts behaviors linked to the fetus' affiliation and interacts with her unborn child [13]. The interest of this concept is that it can describe a particular aspect of the relationship between the mother and her fetus based on conscious representations. These include cognitive representations of the interactions between the mother and her unborn baby, scenarios that reflect maternal ability to allocate physical and emotional characteristics to her fetus (in recognition of the differentiation of the fetus from herself) as well as dreams and expectations about the unborn child. Some behaviors of the mother also reflect this investment such as the changes adopted by women for the well being of their fetus. In uncomplicated pregnancies, it has been shown that prenatal attachment is positively correlated with good medical care during pregnancy [14]. However depressive symptoms during the last trimester of pregnancy is correlated with a lower prenatal attachment [15]. Furthermore, by studying different methods of screening for trisomy 21, a study showed that the persistence of a doubt on fetal prognosis [16] decreases prenatal attachment. Also in high risk pregnancies [17], prenatal attachment would be decreased. In this perspective, the main objective of this study is to evaluate the impact of the announcement of TTTS on prenatal attachment of the mother to both her twins. We will compare the attachment of these patients (TTTS) to those measured in the other twin pregnancies (uncomplicated monochorionic (UMC) and dichorionic (DC) twin pregnancies), at several milestones during pregnancy. The impact of these complications on the attachment between the mother and both her twins, have not yet been evaluated. As secondary objectives, we will seek the existence of a difference in prenatal attachment scores between each of the twins (JA and JB), depending on the attributions given to them in the TTTS management (the donor, the receiver, the bigger, the smaller…).

- The second dimension we assessed is the psychological adjustment of these women, in terms of anxiety, depression and post-traumatic stress disorder (PTSD) in twin pregnancies (complicated or uncomplicated). The stakes are high for a pregnant woman with TTTS. In addition to the psychic reorganization inherent to any twin pregnancy, she has to face the risk of losing her babies throughout the whole pregnancy. This clinical condition comes at the crossroads of different fields of psychology, such as parenthood, psychotraumatism and grief. The traumatism is defined as a perception of the eruption of a life-threatening event. The DSM-V5 specifies that not only witnessing a threatening death experience but also indirectly, learning that a close relative or close friend was exposed to trauma can produce a PTSD. During pregnancy, a threatening death of the babies can have the same effects on the mother.

Even before the announcement of TTTS, the announcement of a twinning early in pregnancy often comes as a shock, thus modifying these parental representations. While the infant was imagined alone, one must now acknowledge the two twins, with all the anxiety, stress and projections that twin pregnancies can stimulate [18]. These psychological difficulties can increase up to 33.3%, the prevalence of major depressive disorders during a twin pregnancy, almost two times higher than single pregnancies. The fear of physical modifications secondary to this pregnancy, as well as the negative ideas concerning twins, are correlated with this depressive dimension [19]. Immediately after this announcement, this pregnancy is classified as a ‘high risk pregnancy’. This label is reinforced by the declaration of monochorionicity, with all its inherent risks and stressful management. The pregnant woman is flooded with a medical follow-up marked by uncertainties and doubts concerning the evolution of this pregnancy. And then there is the announcement of the TTTS when it occurs. Even though the risks of twin pregnancies were previously discussed with the medical staff, the moment of announcement of a TTTS can produce a state of shock for the mother. We aimed to study anxiety, depressive symptoms and post-traumatic stress syndrome in this specific setting and compare it to UMC and DC pregnancies.

## Methods

### Design and participants

All consecutive Dichorionic twin pregnancies (DC) or uncomplicated monochorionic (MC) or complicated monochorionic pregnancies with a Twin to Twin Transfusion Syndrome (TTTS), who consulted at least once at the Necker-Enfants Malades Hospital’s maternity ward between February 2013 to April 2014 were eligible for this study. The protocol was proposed to the pregnant women.

Our exclusion criteria included: patients under 18 of age, monochorionic monoamniotic pregnancies; complicated dichorionic pregnancies; higher order pregnancies (Triplets and more); fetal malformations or chromosomal pathologies; severe maternal psychiatric disorders; predictable monitoring difficulties; non french speaking patients. During the study, patients with abortions or double intra-uterine fetal demise (IUFD) were excluded.

Patients were asked to give their written informed consent. The Institutional Review Board (CERES Comity of ethical review for health research projects Cochin Hospital Paris) approved the study the 10th December 2013 (IRB number 20134500001072) and women gave written informed consent after they received verbal and written information on the study. This protocol did not modify the usual medical and psychological management of these patients.

The patients were asked to complete self-administered questionnaires at key-times during the pregnancy (around 20GW, 26GW, 30GW and 3 months in the post-partum (3MPP)), which will evaluate prenatal attachment (Prenatal Attachment Inventory: PAI), perinatal depression (Edinburgh Perinatal Depression Scale: EPDS) and anxiety (State-Trait Anxiety Inventory: STAI). Questionnaires were exchanged by post, for the patients who pursued their follow-up in another hospital (Table 1).

**Table 1:** Design of the Study.

	_T1—Arrival in the department_	_T2—Announcement of TTTS (ANN)_	_T3—Around 20 GW (20GW)_	_T4—Around 26 GW (26 GW)_	_T5—Around 30GW (30GW)_	_T6–3 Months Post-Partum (3MPP)_
_**Complicated Monochorionic pregnancies (TTTS)**_	_Consent; Socio demographic questionnaire_	_PAI; EPDS; STAI Trait and State_	_PAI; EPDS; STAI-State; **PCLS**_	_PAI; EPDS; STAI-State; **PCLS**_	_PAI; EPDS STAI-State; **PCLS**_	_EPDS; PCLS; STAI–State; **PCLS**_
_**Dichorionic pregnancies (DC)**_	_Consent; Socio demographic questionnaire_		_PAI; EPDS; STAI Trait and State_	_PAI; EPDS; STAI—State_	_PAI; EPDS; STAI—State_	_EPDS; STAI—State_
_**Uncomplicated Monochorionic pregnancies (MC)**_	_Consent; Socio demographic questionnaire_		_PAI; EPDS; STAI Trait and State_	_PAI; EPDS; STAI—State_	_PAI; EPDS; STAI—State_	_EPDS; STAI—State_

**PAI:** Prenatal Attachment Inventory, **EPDS**: Edinburgh Postnatal Depression Scale, **STAI**: State Trait Anxiety Inventory, **PCLS**: Post-Traumatic Checklist Scale

The complicated monochorionic pregnancies with TTTS also received a self-administered questionnaire to evaluate the post-traumatic stress disorder (Post-traumatic Checklist Scale: PCLS) at least one month after the laser for TTTS and afterwards that at the same key-times: on announcement of TTTS (ANN), around 20GW, 26GW, 30GW and 3 months in the post-partum (3MPP). When TTTS is diagnosed around 20 gestational weeks (GW), T2 and T3 are merged for the TTTS group. The chosen "key-times" are related to the medical follow-up. Twenty weeks is the gestational age for the quickening (when the mothers start to perceive fetal movements). TTTS usually occurs and is operated between 20GW and 26GW, we hence chose 26GW as a second milestone. The delivery occurs no later than 34 GW complicated monochorionic pregnancies, to avoid late intra-uterine fetal demise; we hence chose 30 weeks as a last milestone before delivery (especially if we consider the increased risk of premature delivery of these pregnancies). We found that with these time intervals, the evaluation did not weigh too much on the subjects, and preserved a high sensitivity to changes in the scales.

### Outcome measurements

#### Prenatal Attachment Inventory (PAI)

Prenatal attachment can be assessed by self-administered questionnaires. We chose the Prenatal Attachment Inventory which was created by Müller [20]. This author defines the concept of prenatal attachment as a "Unique emotional relationship that develops between mother and fetus". It emphasizes the creation of this emotional relationship during pregnancy. Müller sought to improve the initial tool created by Cranley [13] and suggested to focus on the emotional dimension of conscious representations. We chose this tool because it integrates the affective dimension rather then other self-administered questionnaires that focus more on behavior. Few studies have focused on prenatal attachment in twin pregnancies, but the use of rating scales remains valid with little difference compared to singleton pregnancies [21] [22] [23] and a non significant difference between the two fetuses [22].

We previously coordinated the translation of this scale into French, its validation and publication in 2010 [24]. The Prenatal Attachment Inventory is a self-administered questionnaire of 21 items rated in a Lickert scale from 1 (almost never) to 4 (almost always) exploring different aspects of fetal maternal relationship: Fantasy, Interaction, Affection, Differentiation, Sharing. The scores range from 21 to 84. The average value in our reference population used for the validation of the French translation was of 60 (+/- 9). The Prenatal Attachment Inventory has a unique dimension with no cut-off on the scale [24].

#### Post-Traumatic Checklist Scale (PCLS)

This Post-Traumatic Checklist Scale is a self-administered questionnaire measuring the three major sub-syndromes of post-traumatic stress disorder. It was developed according to the diagnostic criteria of the DSM IV [25]. The scale consists of 17 items assessing the intensity of the 17 PTSD symptoms presented in the DSM IV, listed by the subject on a scale of 1 to 5 (1 = not at all, 2 = a little, 3 = sometimes, 4 = often, 5 = very often). The 17 items can be grouped into three subscales corresponding to the 3 main sub syndromes: repetition (items 1–5); avoidance (items 6–12) and autonomic hyperactivity (items 13–17). The assessment consists of surrounding a number to indicate how the subject has been disrupted in the previous month by the stressful event. The scale offers a threshold at 44 for the diagnosis of PTSD and a possible gradation of the traumatic impact with the Likert scale (rating 1 to 5). French Validation studies have shown good psychometric properties of the scale [26] [27]

#### Edinburgh Postnatal Depression Scale (EPDS)

Edinburgh Postnatal Depression Scale was given to mothers to evaluate their depressive symptoms. This self-administered questionnaire of 10 items is commonly used for the evaluation of post-natal depression [28] and has been validated in French for the postnatal period [29] and for the antenatal period [30]. The 10 items are rated from 0 to 3 so the scale ranges from 0 to 30. We retained 11.5 and 10.5 as the recommended threshold for depression in prenatal period [30] and in postnatal period [29] respectively. This scale is frequently used in longitudinal studies and retest effect is not an obstacle to its repeated use.

#### State Trait Anxiety Inventory (STAI)

The State Trait Anxiety Inventory is a self-questionnaire of 40 items created by Spielberger. Developed in the form X in 1969, it was reformulated in the current Y form by Spielberger in 1983 [31]. This form Y was adapted in french by Bruchon-Schweitzeren in 1990 [32]. It's one of the most used anxiety scales. The questionnaire consists of two 20-item parts, which independently assess the State anxiety (STAI-A) and the Trait anxiety (STAI-B). Each item is scored from 1 to 4 so the score varies from 20 to 80. The internal consistency of the two scales is excellent (a> 0.90). The test-retest reliability is better for Trait anxiety (0.65–0.75) than for anxiety state (.34 to .62). Standards exist in different populations. The mean values found in the French standardization sample are respectively 38.39 +/- 9.69 and 41.75 +/- 8.91 for State anxiety state and Trait in pregnant women. This scale allows comparison of mean scores in groups of patients or to distinguish groups with high anxiety by choosing a cut-off (For Women T score >55 correspond to State score> 41 and Trait score> 47).

### Statistical analysis

Statistical analysis was carried out thanks to the Numbers software. Data were expressed as mean +/- standard deviation or frequencies (percentages).

After checking the normality of the data, Student t tests were performed to compare distributions of quantitative variables between independent groups. Paired Student t tests were performed to compare distributions of quantitative variables between different times in the same group of subjects or between the two twins. Qualitative variables were compared between groups using chi- square test.

## Results

Out of 142 patients who visited our unit during this period, 116 were potentially eligible for participation. The protocol was presented to 83 patients, due to the presence of the researcher on the day of their first visit; all of them accepted to be enrolled.

During the research 2 subjects refused to pursue (1 TTTS and 1 UMC) and 4 were excluded for the lost of their two fetuses, despite the laser intervention. Response rates were 81% à 20GW, 86% at 26GW, 75% at 30GW and 70% at 3MPP, so we obtained 11 to 29 (mean 16.58 +/- 5.61) questionnaires in each group at each time.

### Sample Characteristics

We summarize the socio-demographic characteristics of our population in Table 2. We found a significantly higher length of the couple’s relationship in dichorionic compared to monochorionic pregnancies (p <0.01). Women in dichorionic groups are also significantly older (p <0.001), with higher education/university level (p<0.01). The percentage of pregnancies obtained by ART is significantly higher in DC pregnancies group (74.2% vs 7%) (p <0.001).

**Table 2:** Socio Demographic characteristics.

		Total	Dichorionic pregnancies (DC)	Uncomplicated monochorionic pregnancies (MC)	Complicated monochorionic pregnancies (TTTS)
**N**		83	23	29	31
**Socio-demographic characteristics**	Mother’s age	34 +/- 6 (Min 20 Max 50)	37 +/- 6 ^a^ ^b^ (Min 21 Max 50)	32 +/- 5 ^a^ (Min 21 Max 42)	31 +/- 4 ^b^ (Min 20 Max 39)
	Confirmed support of partner	78 (5 missing Data)	22	26	30
	Length of couple relationship (years)	8.43 +/- 4.23	10.1 +/- 4.93 ^**c**^	6.55 +/- 3.85 ^**c**^	8.095 +/- 3.21
Education level	Uncompleted A-level / Completed A-level / Some University / Completed University	13% / 12% / 25%/ 51%	0% / 7% / 23% ^d^/ 70% ^d^	27% / 8% / 19% ^d’^/ 46% ^d’^	14% / 24% / 33% ^d’^/ 29% ^d’^
**Maternal Medical history**	Obstetrical History (GG, IVG, IMG FC)	38 (45.8%)	18 (58%)	14 (48%)	6 (26%)
	History of psychiatric disorder (Minor Depressive disorder and eating disorder)	4 (4.8%)			
**Pregnancy Characteristics**	Term of Pregnancy at Inclusion	21.33 +/- 3.03	22.55 +/- 3.47	20.22 +/- 1.09	21.00 +/- 3.46
	Spontaneous pregnancy %	55 (67%)	8 (25.8%) ^e^	25 (89.3%) ^e’^	22 (95.7%) ^e’^
	Donated Gametes	10(12%)	10 (32.3%)	0	0
	Pregnancy desire %	96.25	100.00	86.21	91.30
	Gesture	2.23 +/- 1,07	2.45	2.03	2.17
	Para	0.70 +/- 9.95	0.61	0.66	0.87
Adaptation to twin pregnancy	Good / adaptation needed/ Poor	23 (36%) / 21 (32%) / 20 (31%)	14 (52%)^**f**^ / 10 (37%)/ 3 (11%)	4 (21%) ^f’^/ 5 (26%) / 10 (53%)	5 (28%) ^f’^/ 6 (33%) / 7 (39%)
	Embryonic discount on triplets	3	1	1	1
	Life events during pregnancy (Health or social difficulties)	2		1	1
**Birth Characteristics**	Term of delivery	31.99 +/- 2.98	32.61 +/- 3.67	32.07 +/- 2.19	30.78 +/- 2.44
	Intra-uterine fetal demise or abortion (n-number of fetuses)	9 fetuses	0	0	9

^a^ p<0.001

^b^ p<0.001

^c^ p = 0.003

^d VS d’^ p = 0.003

^e VS e’^ p<0.001

^f VS f’^ p = 0.023

The women were asked about their reactions at the announcement of the twinning in the early course of the pregnancy. This announcement was more difficult to integrate for women with monochorionic than for dichorionic pregnancies (p<0.05). This announcement appears to be in the same way easier to integrate in ART pregnancies (p <0.01).

### Differentiation between the two twins

During the medical monitoring of twin pregnancies, the twins are identified JA and JB by the obstetricians (JA most often being the fetus on the right and JB the fetus on the left). We interviewed the women in order to understand how they differentiated their own babies (Table 3). Most of the women differentiated them by their location and movements. When the pregnancies are at risk, with a high medical monitoring, the differentiation is often made according to the obstetrician’s speech. In TTTS, while the medical monitoring differentiated the two twins, the mothers were significantly less able to do so (p = 0.006).

**Table 3:** Differentiation of the twins by mothers.

	Total	Dichorionic pregnancies (DC)	Uncomplicated monochorionic pregnancies (UMC)	Complicated monochorionic pregnancies (TTTS)
Location and/or Movements	43 (52%)	22 (71%) ^a^	14 (48%) ^a’^	7 (30%) ^a’^
Gender	8 (10%)	8 (26%)	Not applicable	Not applicable
Weight / height	10 (12%)	0	5 (17%)	5 (22%)
Named by physician	13 (16%)	0	8 (28%)	5 (22%)
None	9 (11%)	1 (3%) ^b^	2 (7%) ^b^	6 (26%**)** ^**b’**^
	83 (100%)	31 (100%)	29 (100%)	23 (100%)

^a Vs a’^ p<0.001

^b Vs b’^ p = 0.006

If we combine the medical criteria (weight, size, difference by obstetricians) on one side and representational criteria (location, movement) on the other, the distribution of the means used by women to differentiate their baby is significantly different (p<0.01): the DC mothers use more often inner representational means while MC mothers use more means infiltrated by medical discourse.

### Measurements

#### Prenatal Attachment Inventory

PAI results follow a normal distribution. First of all, we checked the difference between the two twins. Mothers filled two PAI interviews, one for each baby at the several times. Differences between JA and JB (Δ = PAI JA—PAI JB) were at ANN Δ = 1.18 +/- 3.19 (p = 0.80), at T3 Δ = -0.07 +/- 1.99 (p = 0.62), at 26GW Δ = 0.62 +/- 2.72 (p = 0.19) and at 30GW Δ = -0.04 +/- 2.81 (p = 0.49). So, we didn’t find any significant difference between JA and JB in any group at these four times. We did not find any significant difference between the donor and the receiver in the TTS group at these four times.

For the following analyses, we used the mean of PAI JA and PAI JB for each woman (Table 4).

**Table 4:** PAI scores.

	ANN	20GW	26 GW	30GW
DC		46.7 +:_ 6.7 (N = 20)	53.3 +/- 9.4 (N = 27)	55.7 +/- 8.1 (N = 29)
UMC		53.3 +/- 9.7 (N = 25)	59.1 +/- 10.3 (N = 23)	61.7 +/- 8.5 (N = 18)
TTTS	50.6 +/- 12.1 (N = 22)	48.1 +/- 10.6 (N = 18)	54.5 +/- 6.3 (N = 15)	55.6 +/- 8.5 (N = 10)
Total Group		49.9 +/- 9.5 (N = 63)	55.6 +/- 9.4 (N = 65)	57.5 +/- 8.7 (N = 57)

**Fig 1:**
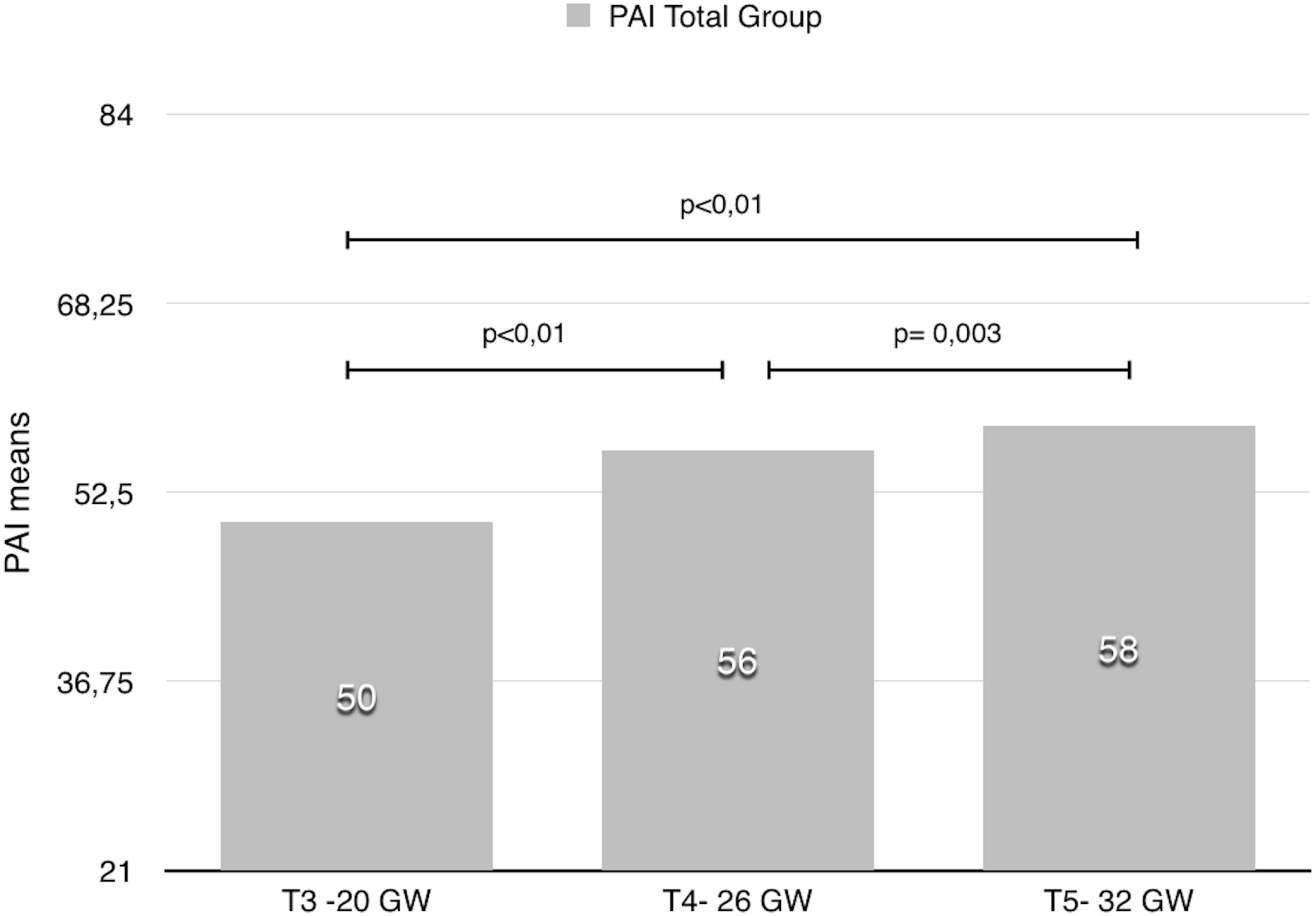
PAI during pregnancy in the Total group.

The Prenatal attachment score were significantly higher at 30 GW than both at 26 GW (p = 0.003) and 20 GW (p<0.001), and at 26 GW than at 20 GW (p<0.001) for the Total group (Fig 1)

**Fig 2:**
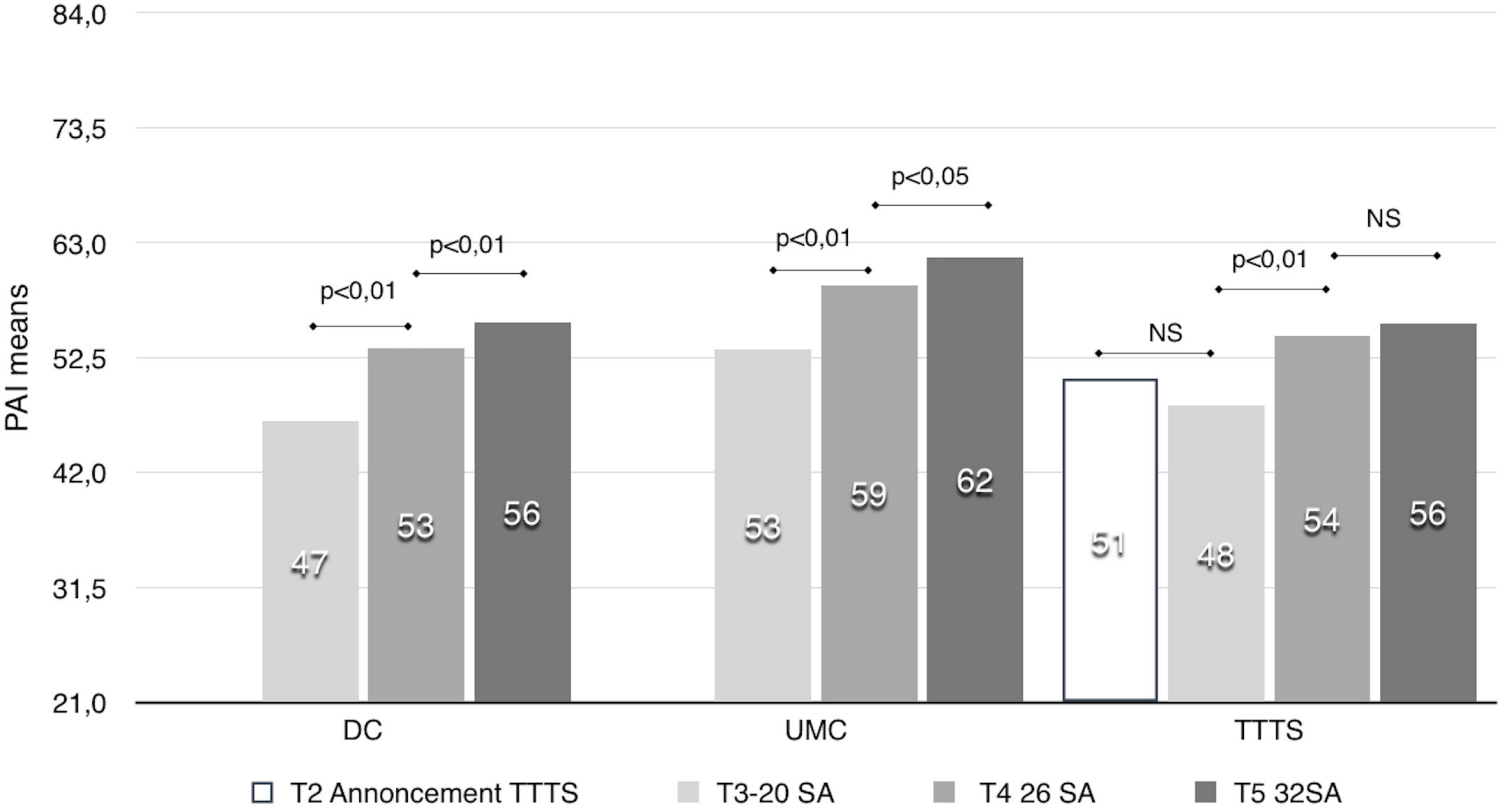
PAI in each of the three groups during pregnancy.

In the DC and UMC groups, PAI scores also increased during pregnancy, but for TTTS group results are not significantly different between 26GW and 30GW (p = 0.95) (Fig 2)

Then, we tested intergroup differences in PAI means.

**Fig 3:**
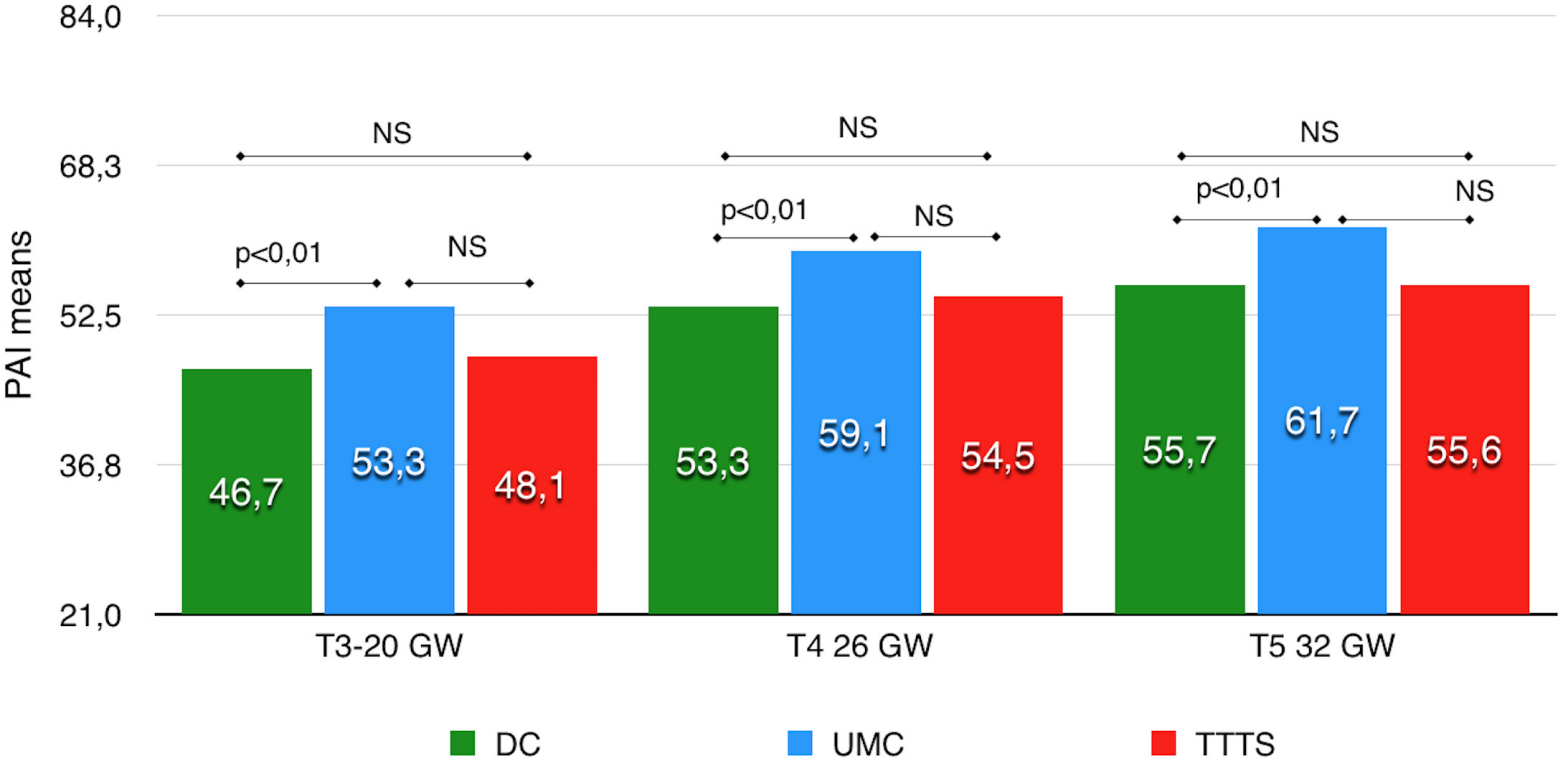
PAI inter-groups.

The prenatal attachment is significantly higher for UMC than for DC at 20 GW (p = 0.006), 26 GW (p = 0.04) and at 30 GW (p = 0.04) (Fig 3). Overall these results showed that the TTTS and DC had non-significant difference in their prenatal attachment score while UMC mothers expressed a significantly higher attachment to their fetuses and expressed it earlier.

#### Post-Traumatic Checklist Scale (PCLS)

Of the 23 subjects included in the study for TTTS, we found at respectively, 20GW, 26GW, 30GW and 3MPP, 20%, 12,5%, 22%, and 18,7% patients with scores above the threshold defined by the scale. So we can point out that 12 to 22% of respondents, depending on the time of pregnancy, have post-traumatic stress symptoms. The three subjects with postnatal scores above the threshold for post-traumatic stress didn't have high state-anxiety scores in early pregnancy, but all three have given birth to premature babies, before 32 weeks. So we found that 30% of mothers with MC pregnancies complicated with TTTS have PTSD at least at one of the four stages.

#### Edinburgh Postnatal Depression Scale (EPDS)

**Fig 4:**
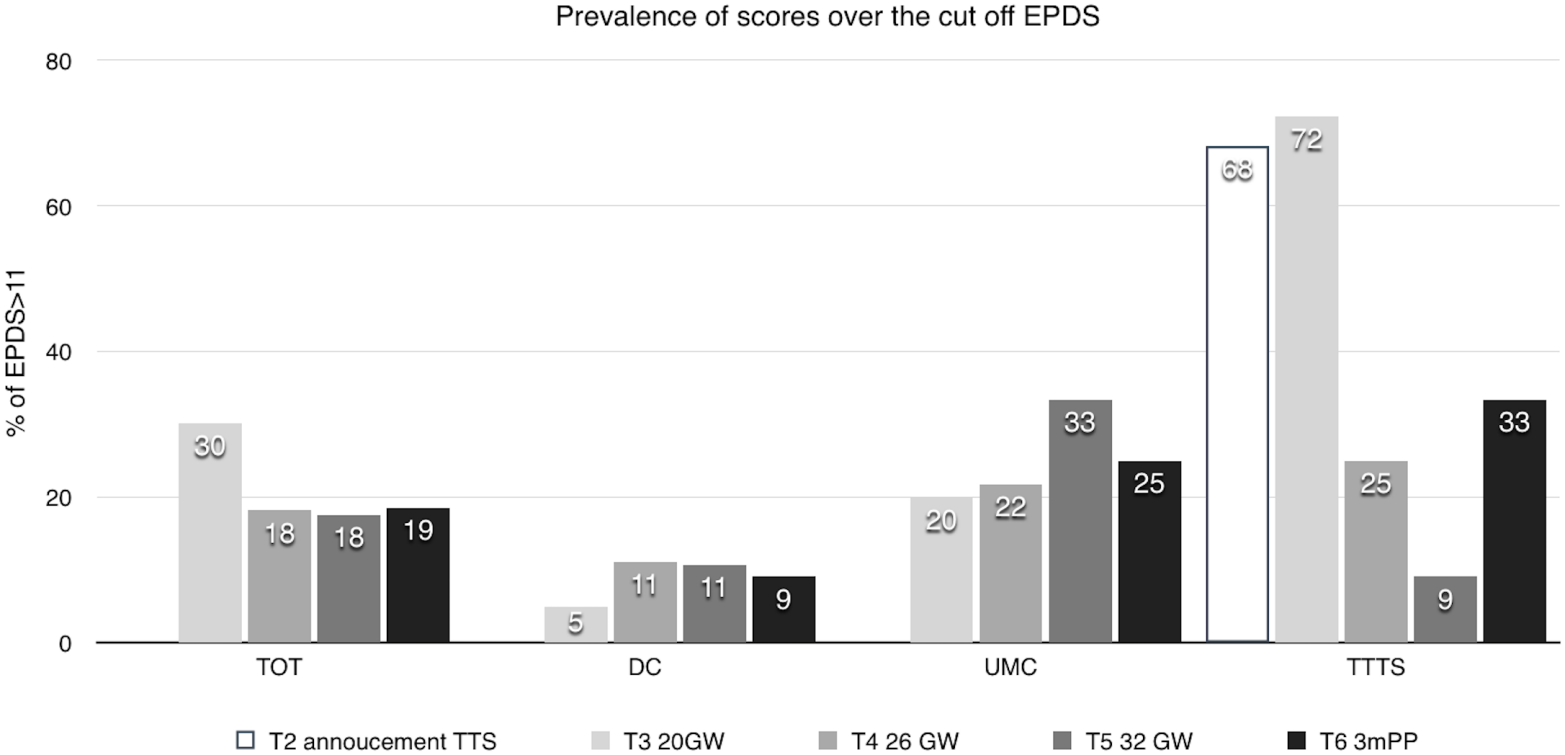
Percentage of EDPS scores over 11.

At 20 GW the mean score at EPDS is statistically higher for TTTS mothers (mean = 12.06 +/-5.58) than for DC (mean = 5.40 +/- 4.64) (p = 0.005), and UMC (mean = 5.68 +/-_ 5.00) (p = 0.007) and the percentage of women with a score above the threshold, set at 11.5, is significantly different in TTS than in UMC (p<0.001) or DC (p<0.001). The percentage of the scores over the cut off is higher in UMC at 30GW than TTTS and DC, but not significant, (p = 0.059). In TTTS at 20 GW, 72% of women have a depressive reaction to the announcement of STT, This percentage is significantly higher than in the other groups p<0.001) (Fig 4).

The percentage of scores over the threshold for all the MC pregnancy (complicated and uncomplicated) is significantly higher than for DC at 20 GW (p = 0.012) but not at the others stages.

When comparing patients who do not exhibit depressive reaction on the EPDS at 20GW after an announcement of TTTS to those that do, we did not find any significant difference in terms of date of announcement of TTTS nor the score of prenatal attachment, nor the adaptation to twin pregnancy. However patients who do not exhibit depressive reaction have lower state anxiety scores (p<0.01), lower Trait anxiety scores (p<0.01) and lower PCLS scores (p<0.01) than those who express a depressive reaction.

In DC pregnancies group, we have put in evidence that at 26GW women presenting a negative score on the EPDS have more frequently conceived their child by ART (p = 0.013). This result does not stand for other stages.

We didn’t find any correlation between the level of prenatal attachment and the EPDS scores.

#### State Trait Anxiety Inventory (STAI)

While the STAI-trait scores are not different between the 3 groups, the means of STAI-State Scores are significantly different (p<0.01) at 20GW between TTTS (mean = 46.2+/-14.4) and DC (mean = 29.5+/-8.7) and UMC (mean = 32.8+/-8.9) (p<0.01). At 26GW, the scores are still significantly different (p<0.05) between TTTS (mean = 41.2+/-14.6) and UMC (mean = 34.4+/-10.6) even though they decrease significantly between 20GW and 26GW for TTTS (p<0.05).

**Fig 5:**
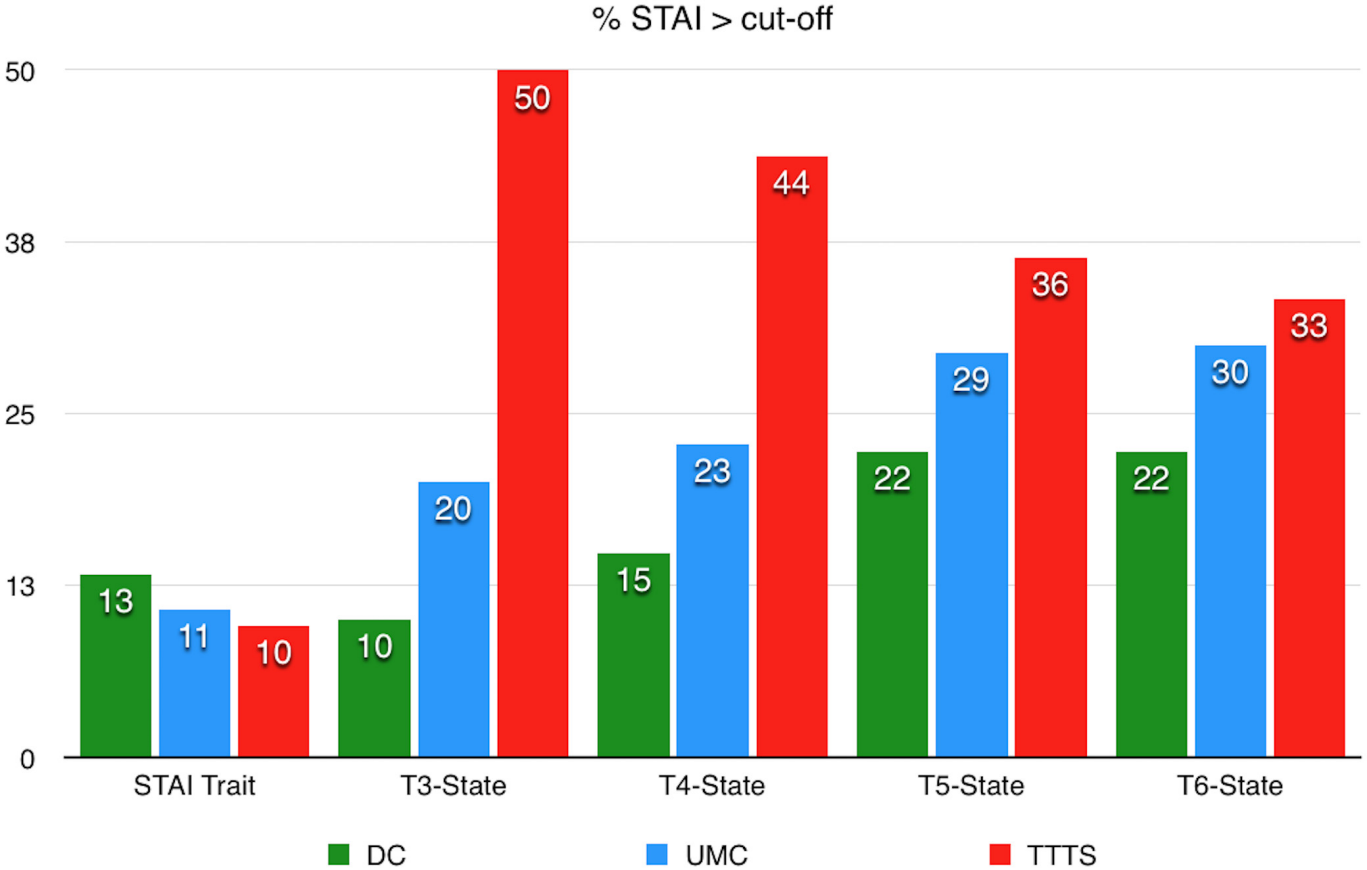
Percentage of STAI scores over the cut-off.

The proportion of subject with a STAI–State over the threshold is significantly higher in TTTS than in DC at 20GW (p = 0.01) and at 26 GW (p<0.05) (Fig 5). This proportion in TTTS group decreases during the pregnancy whereas the STAI-State scores in UMC and DC increase progressively during pregnancy.

We find an association between a positive score to State-anxiety and Trait-anxiety for the Total group at 20GW (p = 0.037), but this association is not confirmed in the TTTS group. For UMC and TTTS the proportion of STAI-state scores over the threshold is still around 30% at 3MPP.

## Discussion

This study is the first to assess, in prenatal period, the maternal emotional experience and the creation of mother-child attachment in uncomplicated and complicated (with TTTS) monochorionic twin pregnancies, which are high-risk pregnancies.

Regarding prenatal attachment, we obtained two significant results. First, we showed that prenatal attachment increases significantly less in the third trimester for mothers in the TTTS group than for DC or UMC groups. We also found that mothers with UMC pregnancies have a higher prenatal attachment than mothers in the DC group. Thus, the increase of prenatal attachment in the last trimester of pregnancy, which is usually described in singleton pregnancies [33] occurs in DC and UMC groups while it does not occur in the TTTS group. We assessed the prenatal attachment at 30 GW, which, for TTTS patients, is 2 weeks before the fetal cerebral MRI and 4 weeks prior to their cesarean section. This period is still a time during which the tests (like cerebral MRI) must confirm the good health of the fetuses. The risk persists for the babies and probably leaves little room for daydreaming, fantasy and anticipation as assessed by the PAI. The burden represented by this uncertainty has been described in other medical circumstances in which the persistence of a doubt can reduce prenatal attachment [16].

Furthermore, we have demonstrated that prenatal attachment was higher in women with UMC pregnancies than in DC pregnancies, and that this difference appears as soon as 20 GW, when the close medical monitoring is instituted for specific monochorionic complications. It has already been shown that repeated ultrasounds during pregnancy, without any pathological context, could increase prenatal attachment [34] [35] [14]. We could be here in a particular context, in which a real concern for the health of the fetus adds up to constant medical attention and repeated ultrasounds. It gives more reality to the presence of the babies. Mothers would feel early concerns for their fetuses, while having to face the risk that a complication occurs. The medical care, the couple and the family environment are more oriented towards the fetuses than towards the pregnancy itself, as is usually described in low-risk pregnancies [11]. The continuing uncertainty of UMC pregnancies could create a particular concern for babies, earlier awareness of the reality and the fragility of their existence that could increase prenatal attachment. Unlike Lawson's study, the risk is known and a medical action is possible if the TTTS occurs. Lawson's study involved a risk of Trisomy 21 in which parents choose to stop the explorations during the pregnancy. Here parents are fully engaged in the medical monitoring of their pregnancy.

We found no significant difference in attachment to each fetus neither when comparing JA and JB, nor for the donor and receiver in TTTS. This lack of difference of prenatal attachment between JA and JB is similar to what has been described in uncomplicated twins [23]. However, in regard to the TTTS group, we found that, the differentiation by the mothers of two babies is less common, while medically it is clear, obvious and repeated at every ultrasound monitoring. This question was asked at the time of inclusion in the study, we verified that there was no significant difference in terms of the inclusion of the three groups, which could have explained the different perceptions. Several hypotheses could be made about this difficulty to differentiate the fetuses, in the TTTS group. It could be a way for the mother to protect the most endangered baby, or be related to the lack of psychic maternal reverie. Indeed, we have shown that in DC pregnancies, internal representations are more often found than medical terms as support to the differentiation of babies.

We have shown that depressive reaction is very common at the time of the announcement of STT, with up to 72% of subjects concerned at 20 GW. The only protective factor found is the existence of a low Trait anxiety. Our study also highlights that prenatal depression rates, as assessed by the EPDS, vary from 17% to 25% in prenatal and 18% post-natal, for the whole group. Post partum depression rate was estimated at 13% in an international meta-analysis [36] while in the French population, one study found a 25% rate in a high risk pregnancy [30] and 18% in a population of low-risk pregnant women [37]. In twin pregnancies, we refer to the international publications that mention a prevalence of 33% of prenatal depression [19], and hence find a lower rate of depressive symptoms in our population [19]. However, details of the three groups are important. In DC pregnancies we found depression rates as low as 5 to 11%. One should also point out that this group is constituted by more than 70% of pregnancies obtained by ART: these awaited pregnancies are hence highly invested by these women. It has been shown that even though twin pregnancies usually generate more anxiety and depression, this effect was lower for twin pregnancies conceived by ART [38]. We also have shown here that the acceptance of a twin pregnancy was easier when it was conceived with ART.

On another hand, the rate of depression is very high for the TTTS group following the announcement of the pregnancy complications but decreases afterwards, to become similar to the other groups: a process of adaptation may be active for these women. Furthermore, we found out anxiety to be also high at the announcement of TTTS. It is possible that the previously described higher rate on the EPDS score might have been generated by the sensibility of the EPDS to anxiety [29] [39]. For the UMC group, the rate of depressive symptoms has a tendency to be higher than in DC group, without reaching statistical significance. It is interesting to examine these results with those of high prenatal attachment in this group. A higher attachment in late pregnancy has been described as protector against the emergence of depressive symptoms [15], in low-risk pregnancies, but also in high-risk pregnancy [40]. Our results are inconsistent with those of Brandons’ study. Two points could explain this inconsistency: whereas their assessment took place between 7 to 38 GW, our results confirmed a rising score on prenatal attachment scale during pregnancy. Furthermore, they mainly studied obstetrical pathologies (Placenta Previa, Toxemia, Multiple Pregnancy Preterm Labor Diabetes, Hypertension Incompetent Cervix, Premature Rupture of Membranes) and found no significant relationship between the severity of the obstetrical risk and mother-fetus attachment. In our series, we show a trend to increased depressive symptoms in UMC, associated with stronger prenatal attachment. We hypothesize that conditions involving close and repeated fetal monitoring increase prenatal attachment with greater objectalisation of the fetus, and more concern for ‘him’. The continuous attention and the important investment brought to these fetuses at risk of developing a disease throughout the pregnancy could lead to a psychic exhaustion, and ultimately a peripartum depression. This theory needs further exploration to be confirmed with bigger sample populations.

Our results show that the TTTS group is marked by a very high anxiety level (50% to 30%) throughout the pregnancy. Whilst the maximum level of anxiety is after the announcement of TTTS, the proportion of anxious women decreases progressively with advancing gestational age. On the contrary, for the other two groups, anxiety tends to increase progressively with advancing gestational age. The anxiety level found in our population is high, and is comparable to the rate of anxiety in singleton pregnancy where a prevalence from 30% [41] to 50% [42] have already been described. Nevertheless, the evolution of anxiety in our three groups differ from the U-shaped curve described in singleton pregnancies with higher levels at the beginning and end of pregnancy [41]. In the TTTS group, we see that it is in the second trimester that the risks will generate more anxiety and then decrease. In the DC group, the significant prevalence of pregnancies achieved by ART may explain the relatively low levels of anxiety, in particular during early pregnancy, as has already been described [42].

Our results show that 30% of women with TTTS will present a post-traumatic stress disorder at least at one time of pregnancy or post-partum. Yonkers et al demonstrated a 5% prevalence of PTSD in the general population of pregnant women [43], regardless of the traumatic event. In a study of perinatal risk in premature births before 33 weeks, maternal PTSD was estimated between 26 and 41% depending on the severity of the perinatal risk [44]. Indeed, the subjects with postnatal scores above the threshold for post-traumatic stress have all preterm birth before 32 weeks. Although the PCLS was studied in reference to the announcement of TTTS as the traumatic event, premature birth could also be a confounding factor

These results should be discussed in light of potential limitations. First, we compared a relatively small number of women. Nevertheless, we had a good response rate, and obtained 11 to 29 questionnaires in each group at each step. The good acceptance rate to the research, the specificity of these pregnancies associated with the rarity of this disease followed in a referral center, support the assumption of our group being a good representative sample of the target population. Second, in the diagnosis of depressive disorders, we only used the EPDS. It is the most sensitive tool to test and retest the evolution of these women to the rhythm of the proposed assessments. The EPDS presents an acceptable sensitivity and the ease of execution needed in the design of our study [45] However, we were unable to confirm this screening by a standardized interview to validate the depressive disorder. In a future study, we should consider adding a tool to better diagnose and classify the depressive disorder detected.

Another potential limitation may have been secondary to the different management of twin pregnancies. While TTTS patients benefit from a systematic psychological interview when they came into the department, the other two groups had psychological follow-up offered but only had this interview at their request. The effect of this interview was not measured here.

For ethical reasons this research is fully integrated into the routine management of patients. However, when we had a concern for the women’s mental health an active management care was made with both specific care and unspecific support.

Although the physicians in charge of the diagnosis, announcement and management of these pregnancies all worked very closely as a team with a common medical procedure and announcement, the personal style and the relationship built with the patients could have an effect on their perception. We did not address this effect in our current study but plan to approach it in future studies.

These results have direct implications in the clinical psychological attention given to each of these groups. The elevated depressive reaction rate in the weeks surrounding the announcement of TTTS, emphasizes the need for very sustained attention to these mothers. The obstetrical team must pay attention to the emotional reaction of the mother; psychological counseling should be offered systematically. When anxiety and depressive reaction are too important, a referral to a psychiatrist specialized in perinatology should be considered to relieve the patient by appropriate prescription of psychotropic medication. For UMC pregnancies, although medical monitoring is intense throughout the pregnancy, the psychological impact is different in this setting. Women, whose pregnancies are finally UMC show a different evolution of their attachment to their fetuses, proving the presence of an underlying psychic activity. The persistent risk of complications, even if they are not confirmed, can lead to mental exhaustion and ultimately a peripartum depression. The medical attention and psychological support must also be offered to the UMC women. Thus, even when the ultrasound results are reassuring, the psychological burden of this follow-up can be very important for those pregnant women; obstetrical teams should take all needed precautions in the peripartum management of these patients. With the consent of the patient, a postnatal psychological follow-up should also be organized systematically for these patients.

We have therefore shown that MC pregnancies experience a different dynamic of prenatal attachment than DC pregnancies. The results presented here are purely quantitative, and open hence a number of questions concerning the representations at work in these women during their pregnancies. A qualitative analysis of interviews, focused on maternal representations is a crucial complement for this work. this will also allow us to explore the psychodynamic processes that women experience throughout these pregnancies and their thoughts and feelings about the utero laser intervention on fetuses. In addition, the longer-term impact on mother-child interactions must also be evaluated in future studies. Indeed prenatal attachment showed a correlation with maternal post partum sensitivity, mother's ability to perceive and verbalize the needs of her child [46]. It will be interesting to see in a future work if the changes of prenatal attachment have consequences in mother-child relationship and to assess the father’s psychological state and the impact of his support to the mother. Finally, a work for the improvement of modalities for the announcement of complications by the physicians would complete the evaluation of these specific pregnancies.
